# Genetic dissection of marker trait associations for grain micro-nutrients and thousand grain weight under heat and drought stress conditions in wheat

**DOI:** 10.3389/fpls.2022.1082513

**Published:** 2023-01-16

**Authors:** Narayana Bhat Devate, Hari Krishna, Chandra Nath Mishra, Karthik Kumar Manjunath, V. P. Sunilkumar, Divya Chauhan, Shweta Singh, Nivedita Sinha, Neelu Jain, Gyanendra Pratap Singh, Pradeep Kumar Singh

**Affiliations:** ^1^ Division of Genetics, ICAR-Indian Agricultural research institute, New Delhi, India; ^2^ ICAR- Indian Institute of Wheat and Barley Research, Karnal, India

**Keywords:** wheat, grain iron, and zinc content, drought and heat stress, GWAS, SNPs

## Abstract

**Introduction:**

Wheat is grown and consumed worldwide, making it an important staple food crop for both its calorific and nutritional content. In places where wheat is used as a staple food, suboptimal micronutrient content levels, especially of grain iron (Fe) and zinc (Zn), can lead to malnutrition. Grain nutrient content is influenced by abiotic stresses, such as drought and heat stress. The best method for addressing micronutrient deficiencies is the biofortification of food crops. The prerequisites for marker-assisted varietal development are the identification of the genomic region responsible for high grain iron and zinc contents and an understanding of their genetics.

**Methods:**

A total of 193 diverse wheat genotypes were evaluated under drought and heat stress conditions across the years at the Indian Agricultural Research Institute (IARI), New Delhi, under timely sown irrigated (IR), restricted irrigated (RI) and late sown (LS) conditions. Grain iron content (GFeC) and grain zinc content (GZnC) were estimated from both the control and treatment groups. Genotyping of all the lines under study was carried out with the single nucleotide polymorphisms (SNPs) from Breeder’s 35K Axiom Array.

**Result and Discussion:**

Three subgroups were observed in the association panel based on both principal component analysis (PCA) and dendrogram analysis. A large whole-genome linkage disequilibrium (LD) block size of 3.49 Mb was observed. A genome-wide association study identified 16 unique stringent marker trait associations for GFeC, GZnC, and 1000-grain weight (TGW). *In silico* analysis demonstrated the presence of 28 potential candidate genes in the flanking region of 16 linked SNPs, such as synaptotagmin-like mitochondrial-lipid-binding domain, HAUS augmin-like complex, di-copper center-containing domain, protein kinase, chaperonin Cpn60, zinc finger, NUDIX hydrolase, etc. Expression levels of these genes in vegetative tissues and grain were also found. Utilization of identified markers in marker-assisted breeding may lead to the rapid development of biofortified wheat genotypes to combat malnutrition.

## Introduction

Wheat (*Triticum aestivum* L.) is one of the most extensively cultivated crops in the world and contributes a major portion of the calories in the global diet. Wheat provides up to 60% of daily energy needs in developing and underdeveloped nations (Wang et al., 2011). Farming of the crop has undergone significant improvements in productivity and production over the past 50 years, under the moniker “green revolution” (Pingali, 2012; Yadav et al., 2019). As a result, we are in a reasonably secure position to satisfy the demand for food. However, a major emphasis was placed on productivity to hasten the food supply rather than giving importance to the wheat’s quality. Compared with naturally occurring wheat, our improved cultivars have a lower level of micronutrients in their grains (Gupta et al., 2021), which is referred to as the “dilution effect” (Murphy et al., 2008; Khoshgoftarmanesh et al., 2011; Amiri et al., 2015).

In areas that are severely affected by micronutrient deficiencies, cereals make up the majority of daily dietary intake. The micronutrient content of staple cereals like wheat and rice, especially the amount of iron (Fe) and zinc (Zn), is not at its optimal level, and milling further decreases this concentration (Rathan et al., 2022). Enhancing the nutritional qualities of crop plants is a strategy known as biofortification that can be used to fight micronutrient deficiencies in food (Bouis et al., 2011). Agronomic biofortification is based on optimized fertilizer applications, whereas genetic biofortification is based on traditional plant breeding and/or genetic engineering to improve nutrient concentrations (Cakmak, 2008; Borrill et al., 2014). Out of these two strategies, genetic biofortification has been identified as an effective and affordable technique to enhance food nutrition content in a sustainable long-lasting way to combat mineral nutrition deficiencies (Krishnappa et al., 2022).

Among the most common human micronutrient deficiencies worldwide, iron and zinc deficiencies are important ones (Welch and Graham, 1999). Hemoglobin, a vital substance for the transportation of oxygen and carbon dioxide, preserves the acid–base balance in the blood and contains a significant amount of iron. Iron deficiency results in anemia, which negatively impacts hemoglobin function and impairs the physical and mental development of children, and can cause maternal mortality in undernourished pregnant women. Zinc promotes growth, controls the body’s immune response, and participates in the production of numerous enzymes (Liu et al., 2014). Inadequate intake of zinc increases the risk of developing cancer, impaired immune function, defective bone formation, and contagious infection (Brown et al., 2004; Gibson, 2006).

The majority of wheat-growing land is affected by sporadic rains, rising temperatures, and heat waves on a regular basis. Heat and drought stress have an impact on grain-filling time, starch accumulation, and seed size. In addition, during heat and drought stress, the grain sink capacity reduces to a greater extent (Zahra et al., 2021). Food and nutritional security will worsen as a result of rising temperatures brought on by climate change and declining water availability in the majority of spring wheat growing regions (Velu et al., 2016). Grain iron and zinc content are complex characteristics affected by multiple genes, with a complicated genetic inheritance, and is significantly influenced by environmental factors (Krishnappa et al., 2017). Drought and heat stress often change the expression of genes involved in nutrient accumulation and translocation owing to polygenic inheritance and genotype-by-environment interactions.

The direct uptake of minerals from the soil and/or the remobilization of minerals held in vegetative tissues during grain filling are the two main sources of grain mineral supply (Kutman et al., 2012). According to research, the vegetative tissues of wheat plants hold the majority of the reserves of grain micronutrients, with more than 70% of these reserves being remobilized during grain filling (Erenoglu et al., 2011; Cu et al., 2020). Abiotic stress influences the vegetative growth and osmotic regulation of plants, indirectly influencing mineral nutrients in the food grains. Heat and drought stress shortens grain-filling durations, decreases starch accumulation, and leads to smaller and shriveled seeds. In addition, heat and drought stress reduce grain sink capacity (Zahra et al., 2021). To breed stable nutrient-containing grain varieties under drought and heat stress, a thorough understanding of the genetic regulation of nutritional traits and its relationship with grain yield is necessary (Samineni et al., 2022). Introgression and mobilization of the genes underlying the desired traits into locally adapted cultivars could result in the genetic improvement of grain nutritional state under abiotic stress.

Recent improvements in high-throughput genotyping and phenotyping have boosted the feasibility of discovering the genetic basis of complex traits (Devate et al., 2022a), such as grain nutrient content. This has led to the popularization of whole-genome-marker-based techniques such as Quantitative trait loci (QTL) mapping, genome-wide association studies (GWASs), and genomic selection (Krishnappa et al., 2021; Anilkumar et al., 2022; Harikrishna et al., 2022). Previous research has been conducted to find grain iron- and zinc- associated QTLs in order to identify potential candidate genomic loci (Xu et al., 2012; Crespo-Herrera et al., 2017; Krishnappa et al., 2017; Velu et al., 2017; Wang et al., 2021). However, QTL mapping requires a long time to establish biparental mapping populations when compared with genome-wide association studies (Edae et al., 2014).

The precise modulation of a complex quantitative trait for adaptation to a specific environmental condition, such as drought or heat stress, necessitates the identification of relevant genetic regions, such as QTLs (Puttamadanayaka et al., 2020; Sunil et al., 2020). GWAS is one of the most effective strategies for identifying genes/QTLs based on linkage disequilibrium (LD). The extent of LD across the genome is greater in self-pollinated crops, such as wheat (Roncallo et al., 2021), providing high resolution and power of association. High-density SNP markers, which are employed in GWASs, may screen large gene pools of breeding material. GWASa have been widely utilized in numerous crops to predict candidate genes using genome-wide-dense markers for various complex traits (Sukumaran et al., 2015; Liu et al., 2018; Srivastava et al., 2020; Alseekh et al., 2021; Danakumara et al., 2021; Devate et al., 2022a; Khan et al., 2022; Tiwari et al., 2022). The benefits of GWASs include the ability to identify Marker trait association (MTAs) with high resolution utilizing diverse germplasm, making the method more efficient and less expensive than biparental QTL mapping (Jin et al., 2016). GWAS is one of the best methods to identify robust QTLs that have an effect in both normal and stress environments (Jamil et al., 2019; Ahmed et al., 2021; Saini et al., 2022). As a result, GWASs have evolved into a potent and widely used method for studying complex traits (Tibbs Cortes et al., 2021).

There have been attempts to identify the genomic regions targeting grain nutrient content such as iron and zin using GWASs (Bhatta et al., 2018; Kumar et al., 2018; Velu et al., 2018; Krishnappa et al., 2022; Rathan et al., 2022). However, all of them are conducted under stress-free ideal conditions for wheat growth, with a few exceptions (Devate et al., 2022b). Most of the wheat-growing area is impacted by drought and heat stress, influencing the quality and quantity of wheat production. Hence, in this study, our focus is on the identification of a candidate genomic region for grain iron and zinc content in wheat grown under drought and heat stress.

## Materials and methods

### Plant material and field experimentation

**Figure 1:**
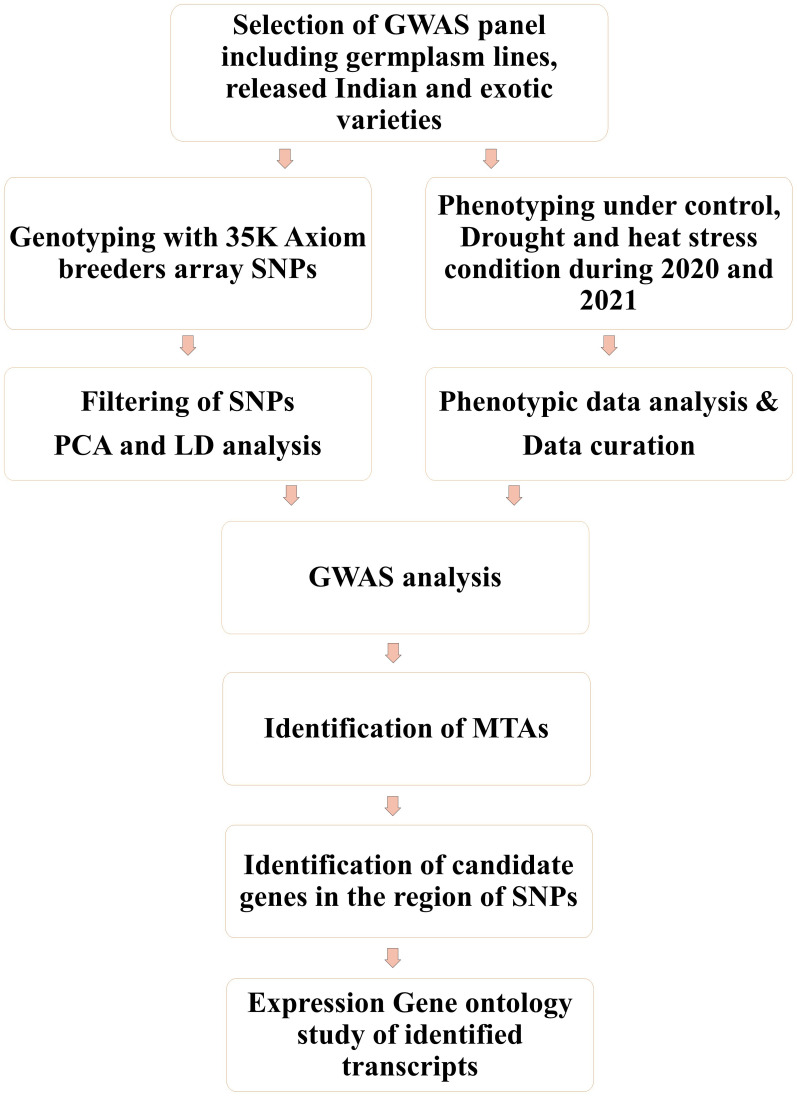
Flow chart of the genome-wide association studies (GWASs) conducted.

The association panel under investigation included 193 genetically diverse bread wheat genotypes (**Supplementary Table 1**) consisting of advanced breeding lines, commercial cultivars, elite varieties, germplasm Core-set, and synthetic derivatives. The plant material collections were available at the Division of Genetics, Indian Agricultural Research Institute (IARI), New Delhi. Field experiments were carried out at the field station of the ICAR–Indian Council Agricultural Research Institute, New Delhi (28.6550° N, 77.1888° E, mean sea level 228.61 m) for 2 years, during the rabi seasons of 2020–2021 and 2021–2022. Weather data during the growing season of wheat (November to March) in 2020–2021 and 2021–2022 are included in **Supplementary Table 2**. In each year genotypes were evaluated under three conditions, that is, irrigated (IR), restricted irrigated (RI), and late sown (LS) to impose stress. Irrigated trials received a total of six irrigations, whereas restricted irrigated trials received only one irrigation (i.e., 21 days after sowing in addition to pre-sowing irrigation) to induce terminal drought stress. Terminal heat stress was introduced by planting the crop (late sown; LS) in the second fortnight of December to expose it to high temperatures during flowering and grain filling, rather than the ideal sowing time, which is the first fortnight of November [followed for the control (IR) and drought (RI) treatments]. The genotypes were evaluated under augmented Augmented -Randomized complete block design (Augmented RCBD) (Federer, 1956; Federer, 1961; Searle, 1965), with 193 genotypes and four checks (HD-3271, HD-3086, HD-3237, and HD-2967) replicated twice in each of the six blocks. Each plot consisted of three 1-m lines sown 25 cm apart. Recommended agronomic practices for the given geographical area were carried out in both years. All four check varieties are popular wheat varieties of the northeastern plain zone where experimentation was conducted. HD 3086 and HD 2967 were recommended varieties for timely sown irrigated conditions, whereas HD 3237 and HD 3271 were recommended varieties for RI and LS conditions, respectively. Furthermore, they are non-segregating homozygous lines, hence suitable to evaluate as checks. For a better understanding of the methods followed in the study, see the flow chart in **Figure 1**.

### Phenotyping

In both years, the collection of grain for grain iron content (GFeC) analysis, grain zinc content (GZnC) analysis, and 1000-grain weight (TGW) estimation was carried out by hand picking 20 random spikes from each line into polythene bags. Hand threshing was done to avoid contamination in machines. GFeC and GZnC were estimated using around 20 g of the grain sample from each genotype. A high-throughput energy-dispersive X-ray fluorescence (ED-XRF) spectrometric, bench-top, non-destructive machine (model X-Supreme 8000; Oxford Instruments plc, Abingdon, United Kingdom) (Paltridge et al., 2012), located at ICAR–Indian Institute of Wheat and Barley Research, Karnal, was used for phenotyping. The concentration of grain iron and zinc was measured in mg/kg. The TGW was measured from manual hand sampling of random representative grains from each genotype. The TGW was recorded in grams using an electronic balance.

### Genotyping

Genomic DNA from leaf samples of all 193 genotypes was isolated with the cetyltrimethylammonium bromide (CTAB) extraction method (Murray and Thompson, 1980) followed by a DNA quality check through 0.8% agarose gel electrophoresis. Genotyping was carried out using the Axiom Wheat Breeder’s Genotyping Array (Affymetrix, Santa Clara, CA, United States) with 35,143 SNPs following standard protocols. Allele calling was carried out using the Affymetrix proprietary software package Axiom Analysis Suite, following the Axiom^®^ Best Practices genotyping work flow (https://media.affymetrix.com/support/downloads/manuals/axiom_analysis_suite_user_guide.pdf). SNPs were filtered, and monomorphic markers and markers with a minor allele frequency (MAF) of 5%, missing data of more than 10%, and heterozygote frequency greater than 50% were eliminated from the study. The remaining 13,947 SNPs were analyzed further.

### Phenotypic data analysis

Analysis of variance, descriptive statistics (mean, SD, range, CV, coefficient of variation H^2^, heritability), and adjusted means of phenotypic data were calculated by year and by treatment (2020–2021 or 2021–2022, and IR, RI, or LS) using the R package ‘augmentedRCBD’ (Aravind et al., 2021). Individual best linear unbiased predictions (BLUP), combined best linear unbiased predictions (BLUP) values of IR, RI, and LS across two seasons (denoted as IR_BLUP, RI_BLUP, LS_BLUP), and overall BLUP (C_BLUP) across the six environments were estimated using ACBD-R software (Rodríguez et al., 2018) with the following model:


Yij = µ + Gen j + Envi + Envi × Genj + Blocki(Envi)+ eij.

Yij = µ+ Blocki + IdCheckj + Genj + Checkj + Envi + Envi × IdCheckj + Envi × Genj + Envi × Checkj + Blocki(Envi)+ eij.

*IdCheck_j_*, *Gen_j_*, and *Check_j_* correspond to the effects of the identifier of checks, the unreplicated genotypes, and the checks that are repeated in each block (*Block_i_*), respectively. *Env_i_* is the effect of *i*th environment and *Env_i_ × Gen_j_, Env_i_ × IdCheck_j_, Env_i_ × Gen_j,_* and *Env_i_ × Check_j_* are the interaction effects. µ is the mean and e is the error component (as described in ACBD-R User Manual, Rodríguez et al., 2018).

Graphical representations of the phenotypic data with frequency distribution and box plot were generated using “ggplot2” package from R software. SNP distribution by chromosome with an SNP density plot was generated using the web tool SR-Plots (https://www.bioinformatics.com.cn/en). Pearson correlation coefficient values among the traits in each environment were also calculated.

### Diversity, linkage disequilibrium, and association analysis

Diversity in the GWAS panel was identified by marker-based principal component analysis (PCA) and neighbor-joining (NJ) dendrogram analysis using Genome Association and Prediction Integrated Tool (GAPIT) v3 (Lipka et al., 2012) and trait analysis by association, evolution, and linkage (TASSEL v5.0) (Bradbury et al., 2007), respectively, with default parameters. For cluster analysis, the distance matrix was calculated from TASSEL v5.0 and the NJ tree file in Newick format was exported to iTOL version 6.5.2 (https://itol.embl.de/) to draw the dendrogram.

Intrachromosomal linkage disequilibrium (LD) was calculated from the pairwise *r* ^2^-value, between markers were calculated from TASSEL 5.0, and the LD decay curve was drawn for each A, B, and D genome along with the whole genome. Pairwise *r* ^2^-values were plotted against the distance in base pairs (BP) to estimate LD block size and the distance at the half-LD decay point was noted (Remington et al., 2001).

A total of 13,947 Filtered SNPs and adjusted means for each environment, BLUPs calculated from the two-season data for IR, RI, and LS (separately and overall), and A total of 13,947 Filtered SNPs and best linear unbiased predictions (BLUPs) across the six environments were used to decipher the associated markers with traits using ‘BLINK’ (Bayesian-information and linkage-disequilibrium iteratively nested keyway) (Huang et al., 2019) under GAPIT v3 in R. In the model, PCA-based population structure was applied as a fixed effect to eliminate the effect of population structure in the analysis. A Q–Q plot was drawn to determine association model fitting, by plotting the expected vs. observed *-log10(p)* values. Marker trait associations (MTAs) in all six environments for GFeC, GZnC, and TGW were found to have a significant *p*-value of< 0.0001. For stringent selection, a Bonferroni correction was applied (*p *= 0.05/total number of markers).

### *In silico*, gene ontology and expression analysis

Associated markers identified using GWAS were subjected to a basic local alignment search tool (BLAST) search using the sequence information of the markers. The BLAST search was carried out using the data web service Ensembl Plants (Yates et al., 2022) (https://plants.ensembl.org/Triticum_aestivum/Tools/Blast) against the bread wheat reference genome IWGSC (RefSeq v1.0). Putative candidate transcripts within a 100-kb flanking region of SNPs were identified by region comparison followed by protein produced (or coded) by them using InterPro Classification of protein families (Blum et al., 2021) (https://www.ebi.ac.uk/interpro/). A further gene ontology study of identified genes was carried out to know their biological process, cellular components, and molecular functions from Expression Atlas (Papatheodorou et al., 2020) (https://www.ebi.ac.uk/gxa/home). Expression analysis of putative candidate genes linked to the identified SNPs was carried out in the Wheat Expression Browser by expVIP (Ramírez-González et al., 2018) (http://www.wheat-expression.com/). Expression of genes in high-level tissue, that is, root, shoot/leaves, spike, and grains, was noted down in the transcript per million (TPM). A heat map based on expression data was generated using the ‘ggplot2’ package in R. Genes with a higher expression level were identified.

## Results

### Phenotypic evaluation

**Figure 2:**
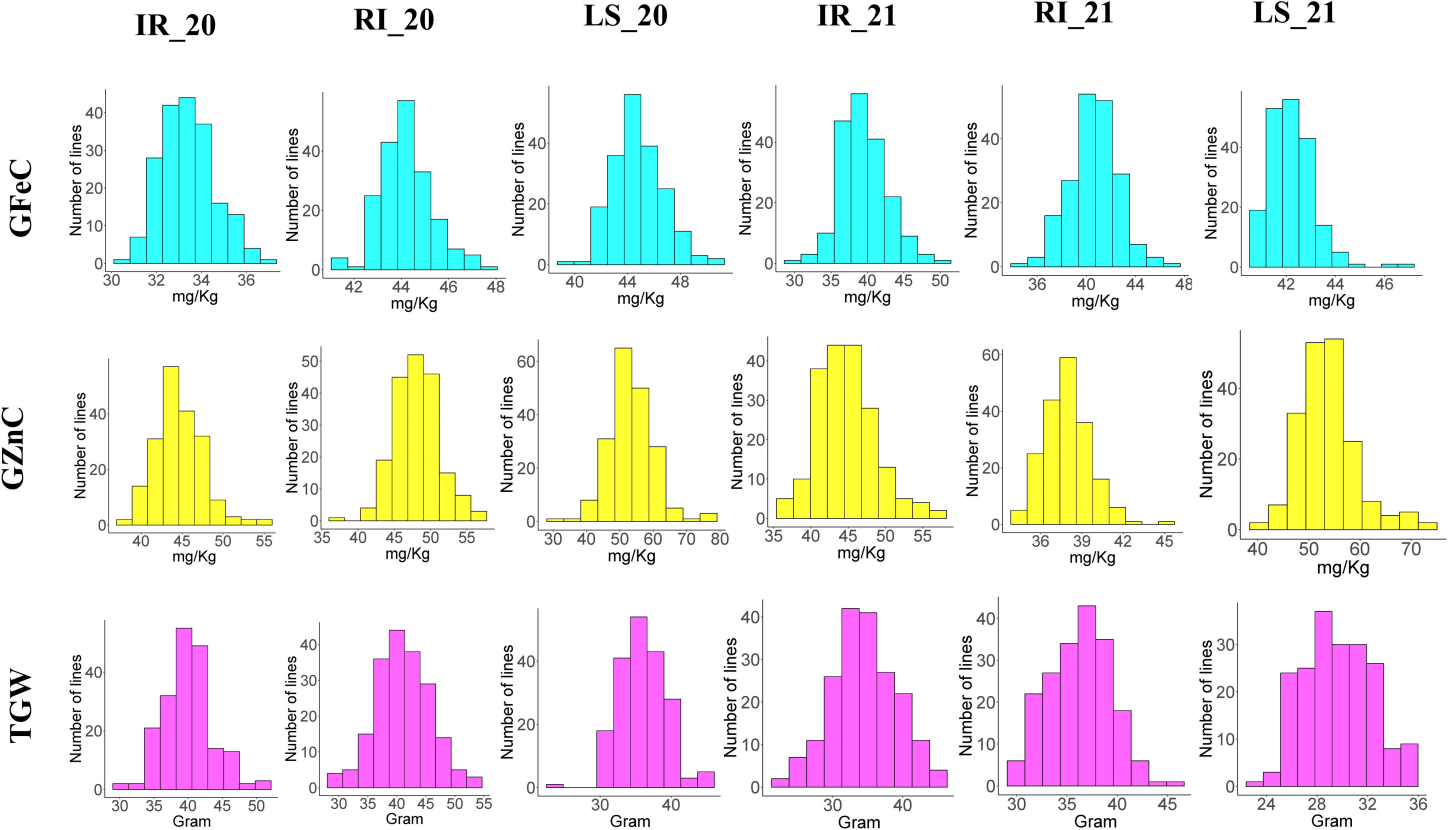
Histogram showing the frequency distribution of all the studied traits across the six environments.

**Figure 3:**
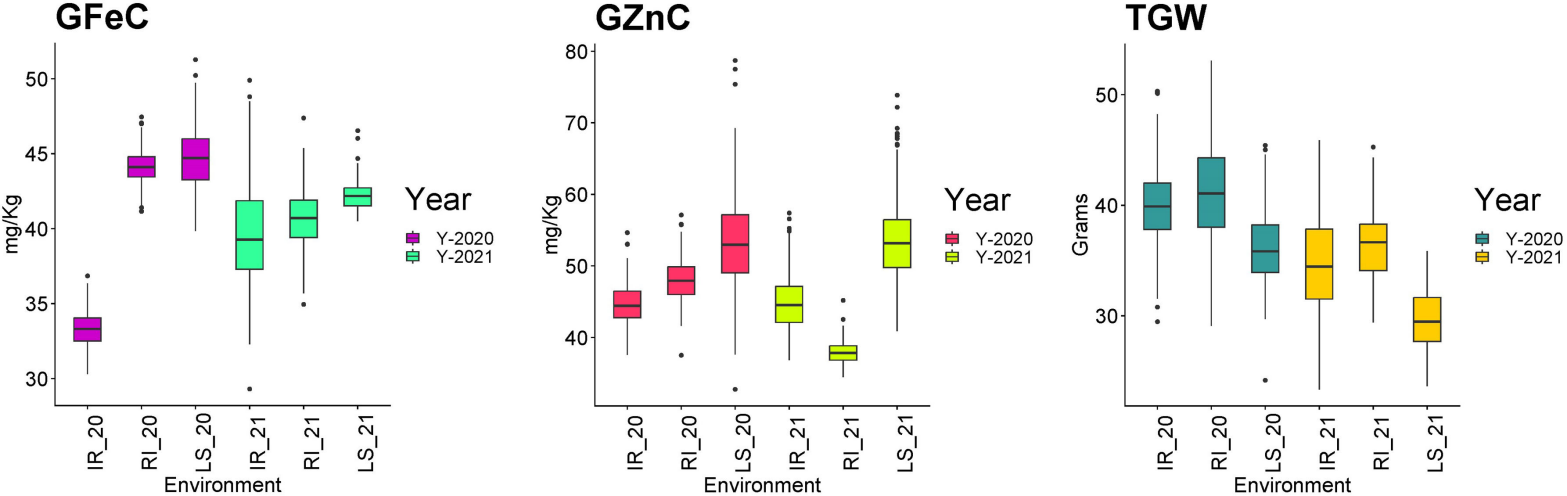
Box plot depicting the distribution of grain iron content (GFeC), grain zinc content (GZnC)p, and the 1000-grain weight (TGW) under timely sown irrigated (IR), restricted irrigated (RI) and late sown (LS) conditions across the years 2020–2021 and 2021–2022.

Descriptive statistics were studied for all three traits, i.e., GFeC, GZnC, and TGW, across IR, RI, and LS treatments from both years and are given in **Table 1**. A frequency distribution histogram demonstrates that all the studied traits were distributed normally in the population (**Figure 2**). Box plots demonstrated that GFeC and GZnC had a higher mean value under RI (Fe 44.29 mg/kg; Zn 48.09 mg/kg) and LS (Fe 44.79 mg/kg; Zn 53.9 mg/kg) treatment than the control (Fe 33.47 mg/kg; Zn 45.13 mg/kg) in the year 2020, and a similar pattern was observed for LS in 2021. Whereas in 2021, under RI, grain iron content (40.87 mg/kg) was higher than the control, but grain zinc content was observed to be lower (39.24 mg/kg). There was a slight increase in TGW upon drought stress, and a significant decrease under heat stress in both the 2020 and 2021 growing periods, compared with control (**Figure 3**).

**Table 1:** Descriptive statistics, analysis of variance (ANOVA) and heritability for GFeC, GZnC, and TGW across the six environments timely sown irrigated (IR), restricted irrigated (RI) and late sown (LS) treatments evaluated in New Delhi during the 2020 and 2021 growing periods.

Trait	Env	Mean ± SD (range)	MSS	CV	hBS
Fe	IR_20	33.47 ± 2.49 (27.01–40.9)	6.4 **	5.28	51.14
	RI_20	44.29 ± 3.65 (35.49–54.59)	14.7 **	5.67	46.54
	LS_20	44.79 ± 3.87 (34.63–57.9)	11.77 **	5.65	56.51
	IR_21	39.57 ± 3.23 (29.3–49.89)	10.2 ^ns^	7.27	19.42
	RI_21	40.87 ± 3.62 (30.34–53.22)	10.25 *	5.14	61.43
	LS_21	42.41 ± 3.19 (35.96–56.87)	11.32 **	6.10	34.88
Zn	IR_20	45.13 ± 5.27 (33.13–63.73)	27.43 **	7.8	55.87
	RI_20	48.09 ± 5.44 (30.64–64.97)	60.02 **	7.11	69.59
	LS_20	53.9 ± 7.77 (30.86–82.92)	37.68 **	4.89	88.89
	IR_21	45.01 ± 6.04 (31.91–65.97)	37.35 **	6.94	74.39
	RI_21	39.24 ± 6.75 (24.98–68.67)	97.03 **	12.64	41.57
	LS_21	53.82 ± 9.42 (32.64–87.69)	40.82 *	9.91	71.38
TGW	IR_20	40.16 ± 5.18 (24.61–55.11)	31.19 **	7.31	72.88
	RI_20	41.67 ± 6.03 (25.36–58.11)	23.5 **	6.72	79.19
	LS_20	35.95 ± 4.82 (19.14–50.02)	37.12 **	7.07	72.37
	IR_21	34.91 ± 5.95 (20.25–50.53)	37.06 **	7.9	80.08
	RI_21	36.58 ± 4.7 (26.19–49.99)	27.38 **	7.49	66.47
	LS_21	29.91 ± 4.88 (19.04–41.28)	22.09 **	11.46	58.13

* p<0.05; **p< 0.01; ns, non-significant. Env, environment; SD, Standered deviation; MSS, Mean sum of squares; CV, coefficient of variation; hBS, heritability broad sense.

Analysis of variance (given as a MSS "Mean sum of squares" ; **Table 1**) demonstrated that all the studied traits in all the treatments across the years showed significant variation, except for GFeC under IR in 2021. The coefficient of variation (CV) ranged from 4.89% to 12.64% for GZnC under LS in 2020 and RI in 2021, respectively. For GFeC and TGW the CV ranged from 5.14% to 7.27% and 6.72% to 11.46% respectively. All the studied traits depicted medium-to-high broad sense heritability, with the highest heritability by GZnC under LS in 2020. The range of heritability of GFeC, GZnC, and TGW was 19.42% to 61.43%, 41.57 to 88.89%, and 58.13% to 80.08%, respectively (**Table 1**).

The Pearson correlation coefficient among the traits in each treatment across the year demonstrated a significant positive correlation between GFeC and GZnC under all environments (*p*<0.001), except under RI in 2020, which showed a non-significant correlation. The correlation of TGW with GFeC and GZnC was non-significant in 2020 under all three treatments. However, in 2021, TGW showed a positive correlation with GFeC under the IR and RI conditions, and a negative correlation with GZnC under IR and LS (**Table 2**). Correlation among the observations across the three treatments and the years for each trait separately demonstrated all positive correlations with high correlation coefficient values for TGW (**Supplementary Figure 1**).

**Table 2:** Correlation among the traits of grain iron content (GFeC), grain zinc content (GZnC), and 1000-grain weight (TGW) in each environment.

Treatment	Traits	Season 2020–21 rabi			Season 2021–22 rabi		
		GFeC	GZnC	TGW	GFeC	GZnC	TGW
IR	GFeC	1	0.24***	0.13	1	0.49***	0.21**
	GZnC		1	–0.02		1	–0.17*
	TGW			1			1
RI	GFeC	1	0.07	0.08	1	0.40***	0.21**
	GZnC		1	–0.04		1	0.03
	TGW			1			1
LS	GFeC	1	0.30***	0.11	1	0.37***	0.12
	GZnC		1	0.1		1	–0.29***
	TGW			1			1

Significant correlations are given in bold text, *** p < 0.001, **P < 0.01, *p < 0.05.

### SNP markers distribution, population diversity and linkage disequilibrium

**Figure 4:**
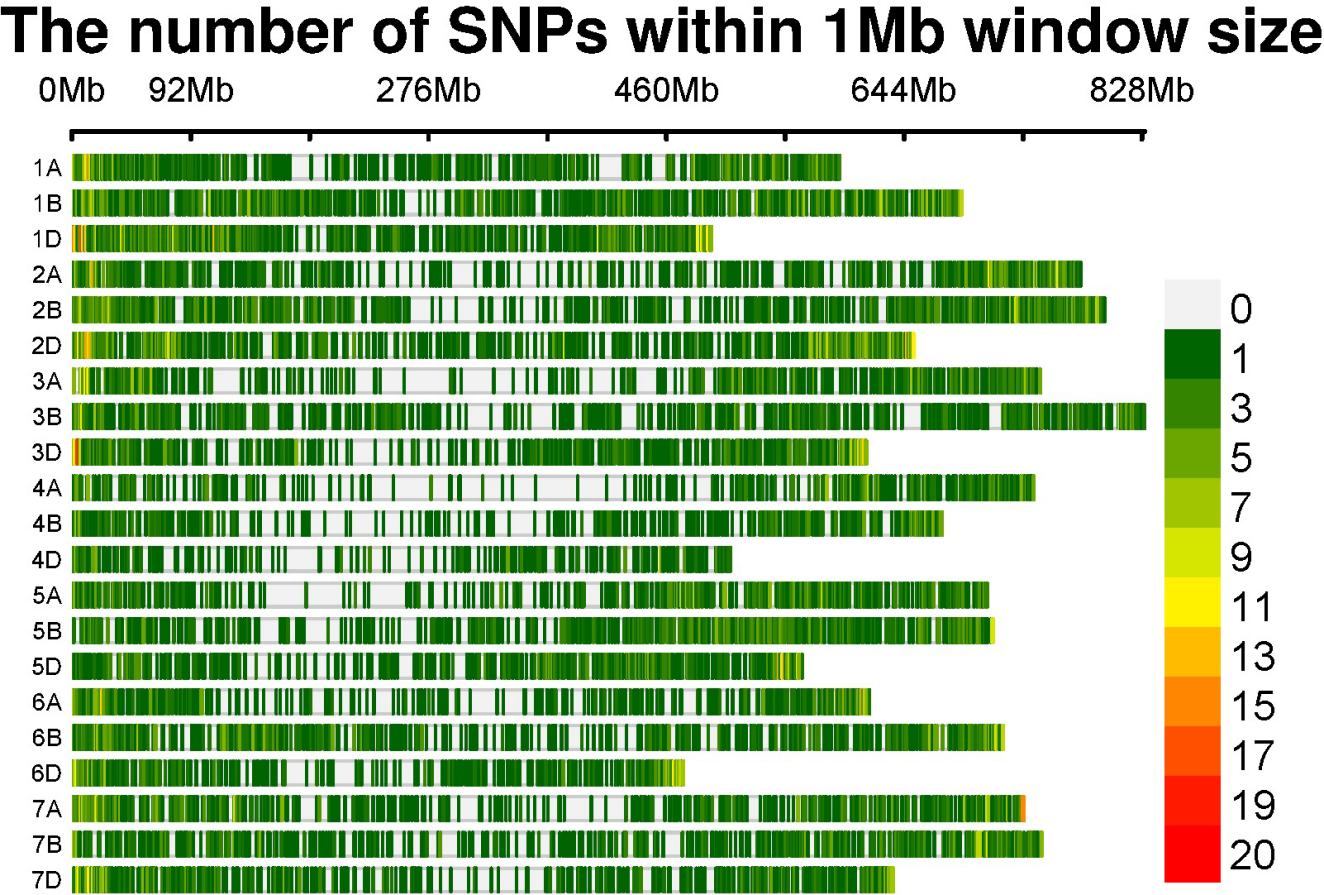
SNP density plot indicating the distribution of filtered SNPs across chromosomes.

Out of the 35,143 SNPs from the 35K array, a total of 13,947 genome-wide SNPs were retained after filtering for a MAF of<0.05, a heterozygote frequency of<0.5, and missing data<0.1 for quality processing. These SNPs were distributed over the genomes A, B, and D, with 4,307, 5,246, and 4,394 SNPs, respectively. The numbers of SNPs distributed over each chromosome are given in **Table 3** and are graphically depicted through an SNP density plot (**Figure 4**).

**Table 3:** Distribution of 13,947 filtered SNPs over chromosomes and three subgenomes A, B, and D.

Chromosome	Subgenome			
	A	B	D	
1	687	998	890	
2	707	927	920	
3	623	717	626	
4	446	435	274	
5	623	762	609	
6	495	727	463	
7	726	680	612	
Total number of SNPs	4,307	5,246	4,394	13,947

**Figure 5:**
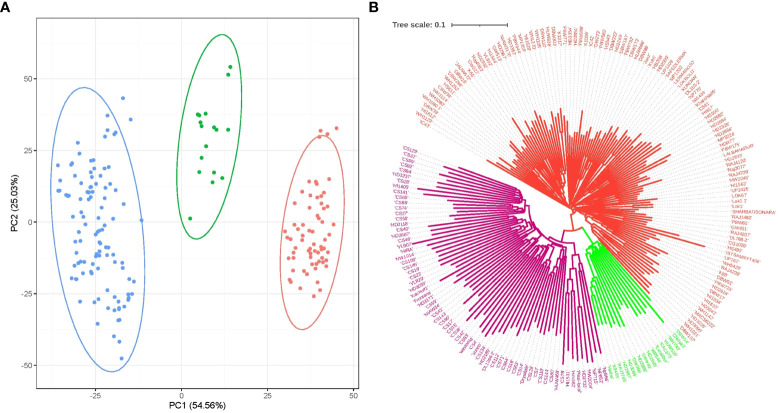
Population groupings in the GWAS panel from different models. **(A)** Principal component-based grouping based on PC1 vs. PC2; and **(B)** neighbor-joining tree based on a distance matrix.

Molecular marker-based PCA demonstrated that PC1 and PC2 corresponded to 54.56% and 25.03% of the variation, and the population was grouped into three subgroups (**Figure 5**). A neighbor-joining dendrogram drawn based on the distance matrix among the genotypes from the GWAS panel inferred three main clusters branching into many clusters in the population (**Figure 5**). PCA-based population grouping was used as a covariate in association analysis to avoid false associations occurring as a result of the population structure.

**Figure 6:**
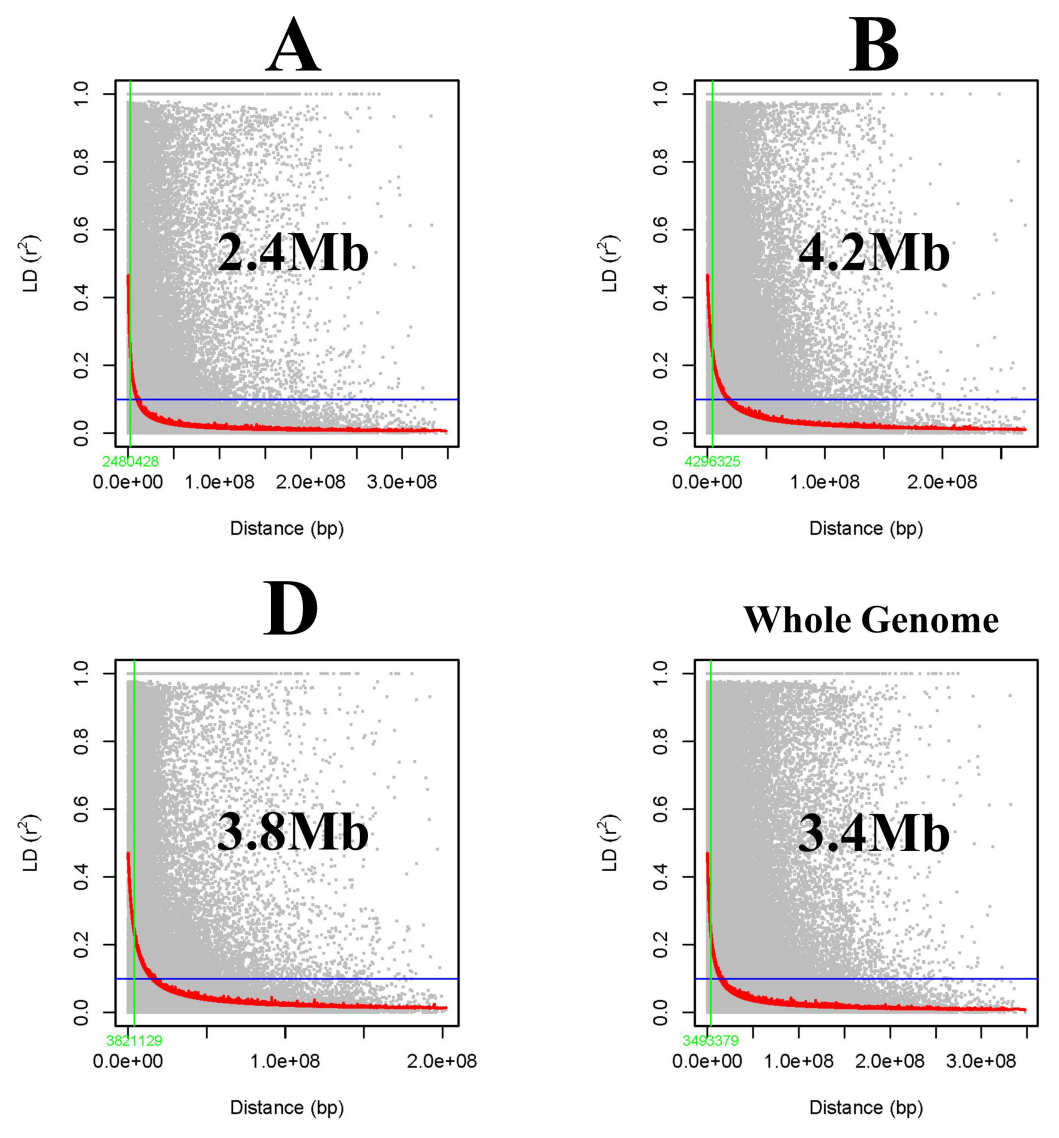
Subgenome **(A, B, D)** and whole-genome-wide linkage disequilibrium (LD) decay in the GWAS panel.

Linkage disequilibrium (LD) between the marker pairs was calculated as *r* ^2^. The LD decay plot was drawn using the *r* ^2^-value against genetic distance in base pairs (bps). A large LD block size of 3.49 Mb was observed for the whole genome indicating that SNPs at this block act as inheritance blocks, whereas subgenomes A, B, and D had block sizes of 2.48 Mb, 4.29 Mb, and 3.82 Mb, respectively (**Figure 6**).

### Genome-wide association study

**Figure 7:**
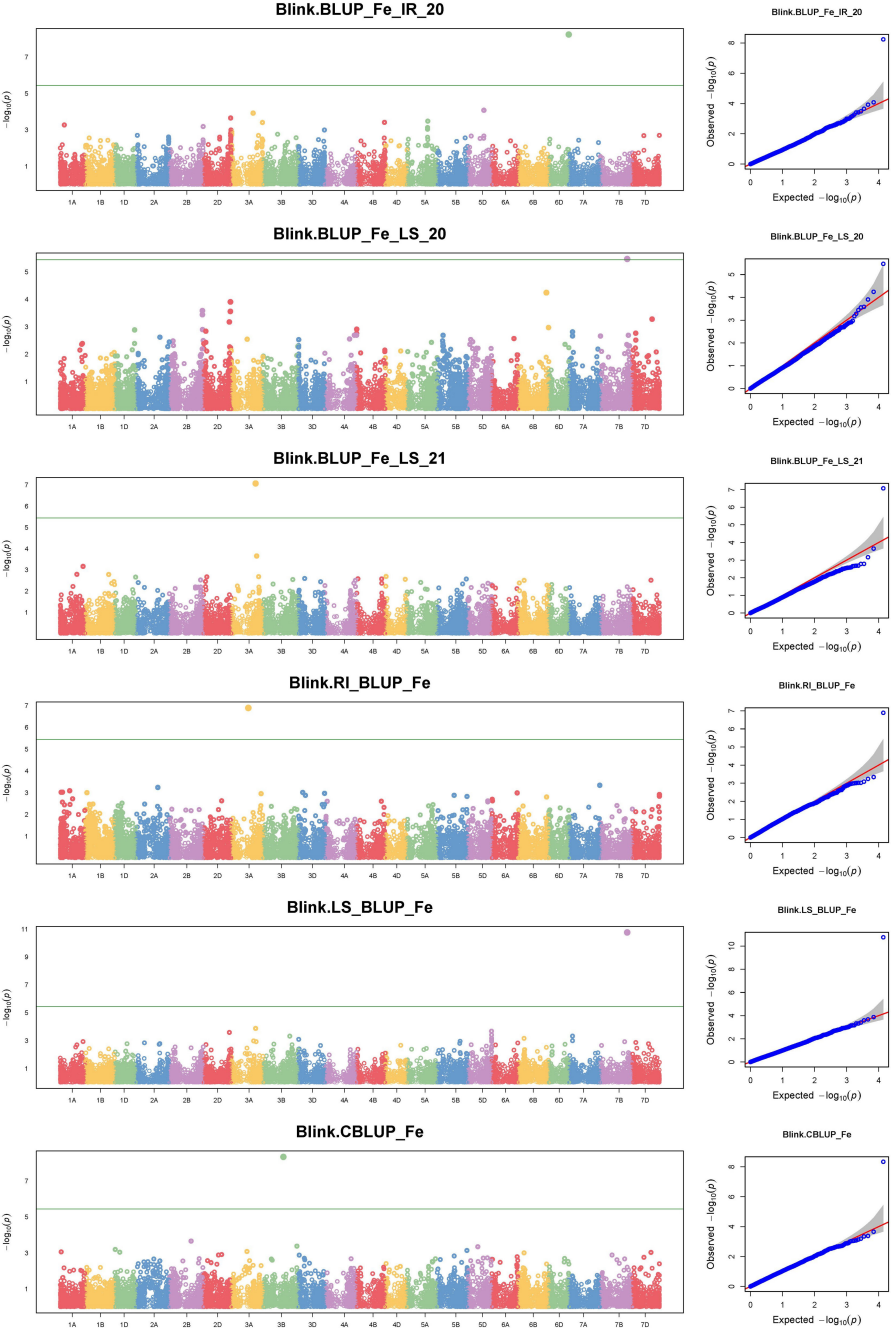
Manhattan and respective quantile–quantile **(Q–Q)** plots of significant associations for GFeC under IR, RI, LS, and combined BLUPs.

**Figure 8:**
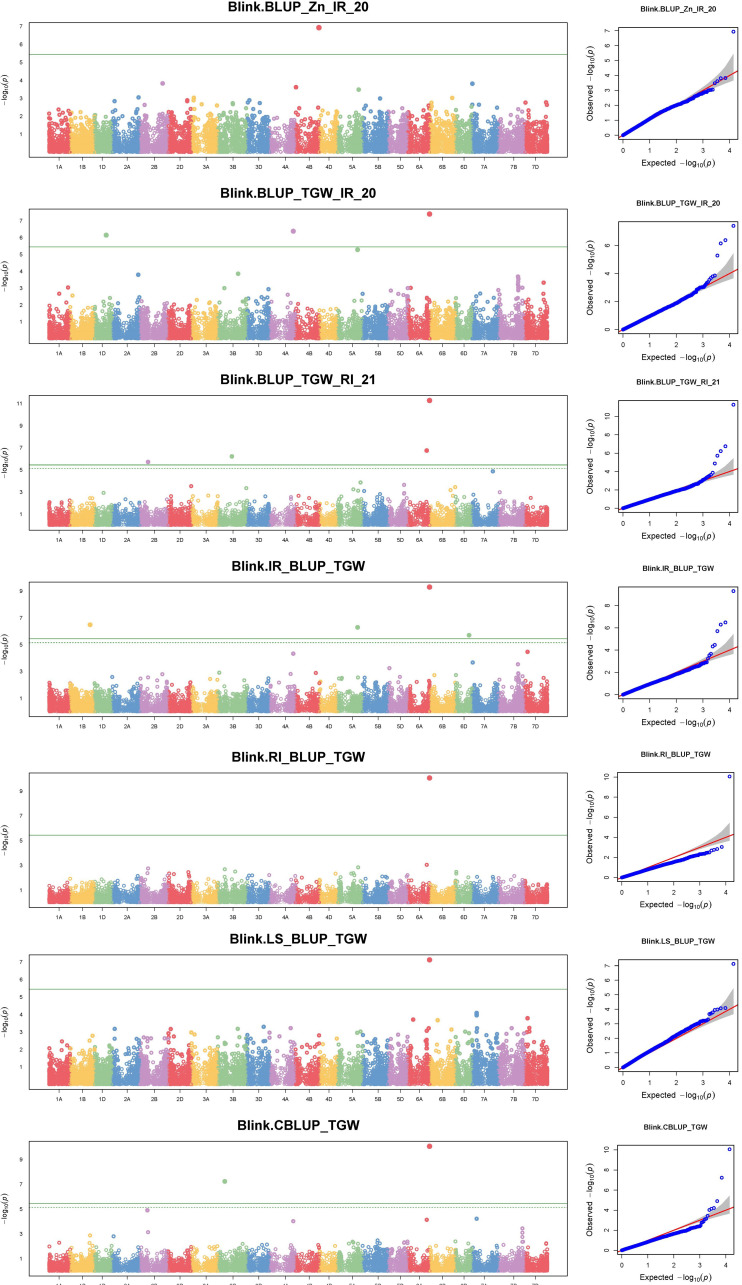
Manhattan and respective Q–Q plots of significant associations for GZnC and TGW under IR, RI, LS, and combined BLUPs.

**Figure 9:**
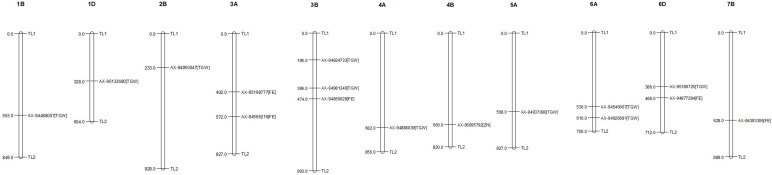
Distribution and position (in Mb) of identified marker trait associations (MTAs) at their respective chromosome with associated trait.

The GWAS for all three traits, with a BLUP value for each treatment in both of the years separately, across all the years, and the overall BLUP, revealed 36 unique marker trait associations (MTAs) for all three traits put together at the cut-off *p*-value of<0.0001. Among them, five, six, and 10 unique, treatment-specific SNPs were linked to GFeC, GZnC, and TGW, respectively. Similarly, based on combined BLUPs, five SNPs were linked to GFeC, and 10 were linked to TGW (**Supplementary Table 3**). To enhance the stringency of the selection a Bonferroni correction was applied (–log_10_(*p*) *>5.45*) and 16 MTAs (five for GFeC, one for GZnC, and 10 for TGW) were retained and they were depicted with Manhattan and quantile–quantile (Q–Q) plots (**Figures 7** and **8**). SNP AX-94926681 located on 6A at 610.4 Mb was linked with TGW, and was identified under more than one treatment and also under combined BLUPs. Similarly, SNP AX-94393306 was linked with GFeC under LS in 2020 and LS_BLUP across the years. Only one linked SNP AX-95095792 was located on chromosome 4B at 660.4 Mb and was identified for GZnC under IR in 2020. A total of five different SNPs were linked to GFeC and were identified on four different chromosomes: 6D, 3A, 7B, and 3B (**Table 4**; **Figure 9**).

**Table 4:** Significant marker trait associations (MTAs) with a Bonferroni-corrected *p*-value (–log_10_(*p*) > 5.45) for traits under study at each environment.

Trait	Environment	SNP	Chromosome	Position	*p*-value	–log_10_(*p*)
**Fe**	IR_20	AX-94877284	6D	4.68E+08	5.77E-09	8.238789
	RI_ BLUP	AX-95168777	3A	4.02E+08	1.29E-07	6.890502
	LS_20	**AX-94393306**	7B	6.28E+08	3.39E-06	5.470092
	LS_21	AX-94565216	3A	5.72E+08	8.49E-08	7.071115
	LS_BLUP	**AX-94393306**	7B	6.28E+08	1.74E-11	10.75943
	C_BLUP	AX-94850629	3B	4.74E+08	4.63E-09	8.334826
**Zn**	IR_20	AX-95095792	4B	6.6E+08	1.17E-07	6.929969
**TGW**	IR_20	**AX-94926681**	6A	6.1E+08	3.96E-08	7.401876
		AX-94888039	4A	6.82E+08	4.15E-07	6.381996
		AX-95133680	1D	3.28E+08	7.11E-07	6.148171
	IR_BLUP	**AX-94926681**	6A	6.1E+08	5.05E-10	9.296763
		AX-94488007	1B	5.63E+08	3.29E-07	6.483375
		AX-94937080	5A	5.68E+08	5.12E-07	6.290645
		AX-95189725	6D	3.86E+08	1.98E-06	5.702482
	RI_21	**AX-94926681**	6A	6.1E+08	5.35E-12	11.27166
		AX-94546067	6A	5.3E+08	1.79E-07	6.746621
		AX-94981340	3B	3.96E+08	6.07E-07	6.217013
		AX-94950047	2B	2.33E+08	1.90E-06	5.720823
	RI_BLUP	**AX-94926681**	6A	6.1E+08	8.73E-11	10.05904
	LS_BLUP	**AX-94926681**	6A	6.1E+08	7.50E-08	7.125002
	C_BLUP	**AX-94926681**	6A	6.1E+08	8.56E-11	10.06758
		AX-94824733	3B	1.95E+08	5.82E-08	7.235218

MTAs identified more than once are given in bold text.

### *In silico* analysis

A total of 16 MTAs were retained after stringent selection and were further searched for their candidate genes in a 100-kb flanking region using sequence information from the SNPs identified with the BLAST search in Ensembl Plants. The SNP AX-94393306, which was present on chromosome 7B, was linked to GFeC and was BLASTed to gene codes for HAUS augmin–like complex subunit 2. A gene ontology study found that the gene was an integral part of the microtubule organizing center, as it plays a role in spindle assembly during cell division. In a 100-kb flanking region of this SNP, there is another transcript coding for the P-loop-containing nucleoside triphosphate hydrolase. Similarly, SNP AX-94850629 was within the region coding for a serine–threonine/tyrosine-protein kinase, which is a catalytic domain with a major role in protein phosphorylation. Two other transcripts coding for the glycoside hydrolase superfamily and the bifunctional inhibitor/plant lipid transfer protein/seed storage helical domain were present in the flanking region of the SNP. Only one MTA AX-95095792 was found to be linked to GZnC at a –log10(*p*) value of 6.92. This MTA was present in the transcribing region codes for restriction endonuclease type II-like protein. In the flanking region of this SNP another two transcribing regions (TraesCS4B02G378800, TraesCS4B02G378500) were identified as coding for cyclophilin-type peptidyl-prolyl cis–trans isomerase domain and tRNA/rRNA methyltransferase, with them having a potential role in protein peptidyl-prolyl isomerization and RNA processing, respectively. A stably identified SNP AX-94926681, linked to TGW, was present in the region coding for ribonuclease H, exonuclease, and RNase T/DNA polymerase III, all of which have crucial roles in nucleic acid binding. Similarly, the location of the transcripts within or near the region of identified MTAs were searched, and are presented in **Table 5**. Two MTAs namely, AX-94950047 and AX-94824733, were located in the non-genic region.

**Table 5:** Putative candidate genes in the 100-kb region of the linked marker with protein produced and gene ontology studies.

Trait	SNP	Chromosome	TrasID	SNP position on the gene	Protein	Gene ontology		
						Biological process	Cellular component	Molecular function
GFeC	AX-94877284	6D	TraesCS6D02G394700	Exon 3	P-loop-containing nucleoside triphosphate hydrolase			
			TraesCS6D02G394800	Flanking	Synaptotagmin-like mitochondrial-lipid-binding domain	Lipid transport	Integral component of membrane	Lipid binding
	AX-94393306	7B	TraesCS7B02G365000	5’ UTR	HAUS augmin–like complex subunit 2	Spindle assembly	Microtubule organizing center organization	
			TraesCS7B02G365100	Flanking	P-loop-containing nucleoside triphosphate hydrolase	ADP binding		
	AX-94565216	3A	TraesCS3A02G326700	5’ UTR	Protein coding			
			TraesCS3A02G326600	Flanking	Di-copper center-containing domain superfamily			
	AX-94850629	3B	TraesCS3B02G295000	Exon 1	Serine–threonine/tyrosine-protein kinase, catalytic domain	Protein phosphorylation	ATP binding/polysaccharide binding	Protein kinase activity
			TraesCS3B02G294900	Flanking	Glycoside hydrolase superfamily	Protein phosphorylation,	ATP binding/hydrolyzing *O*-glycosyl compounds	Hydrolase activity/carbohydrate metabolic process/protein kinase activity
			TraesCS3B02G294800	Flanking	Bifunctional inhibitor/plant lipid transfer protein/seed storage helical domain superfamily	Lipid transport		
	AX-95168777	3A	TraesCS3A02G219000	Intron 1	C-5 cytosine methyltransferase	Negative regulation of gene expression/epigenetic	Nucleus	Methyltransferase activity
GZnC	AX-95095792	4B	TraesCS4B02G378700	Exon 1	Restriction endonuclease type II like			
			TraesCS4B02G378800	Flanking	Cyclophilin-type peptidyl-prolyl cis–trans isomerase domain	Protein peptidyl-prolyl isomerization		Peptidyl-prolyl cis–trans isomerase activity
			TraesCS4B02G378500	Flanking	tRNA/rRNA methyltransferase, SpoU type	RNA processing	RNA binding	RNA methyltransferase activity
TGW	AX-94926681	6A	TraesCS6A02G402600	5’UTR	Ribonuclease H-like superfamily	Nucleic acid binding	Integral component of membrane	
					Exonuclease, RNase T/DNA polymerase III			
	AX-94888039	4A	TraesCS4A02G409100	Exon 4	Chaperonin Cpn60/GroEL	ATP binding	Cytoplasm	Protein folding and re folding
			TraesCS4A02G409000	Flanking	Zinc finger, FYVE/PHD-type	Metal ion binding	Nucleus	Chromatin organization
	AX-95133680	1D	TraesCS1D02G238600	Exon 5	*S*-adenosyl-*L*-methionine-dependent methyltransferase	Methyltransferase activity		
			TraesCS1D02G238700	Flanking	Conserved oligomeric Golgi complex subunit 5	Intra-Golgi vesicle-mediated transport	Golgi transport complex	
			TraesCS1D02G238500	Flanking	DNA-directed RNA polymerase, insert domain superfamily	Transcription, RNA polymerase I and III activity		DNA binding/protein dimerization activity
	AX-94546067	6A	TraesCS6A02G296400	3’UTR	NUDIX hydrolase domain	Hydrolase activity		
	AX-94981340	3B	TraesCS3B02G248500	Exon 15	ABC transporter-like, ATP-binding domain	ATPase activity	ATP binding/membrane	Coupled to transmembrane movement of substances
	AX-94488007	1B	TraesCS1B02G336000	Intron 7	Secretory carrier membrane proteins (SCAMPs)	Protein transport	Integral component of membrane	
	AX-94937080	5A	TraesCS5A02G367700	Exon 4	Protein kinase domain	Protein phosphorylation		ATP binding/protein kinase activity/protein binding
			TraesCS5A02G367600	Flanking	UDP-3-*O*-[3-hydroxymyristoyl] glucosamine *N*-acyltransferase LpxD	Lipid A biosynthetic process		*N*-acyltransferase activity
	AX-95189725	6D	TraesCS6D02G277900	Exon 3	Ankyrin repeat-containing domain superfamily	Protein binding	Plasma membrane	
			TraesCS6D02G278000	Flanking	Pentatricopeptide repeat	Protein binding		
			TraesCS6D02G278100	Flanking	Zinc finger, RING type		Integral component of membrane	
	AX-94950047	2B	NA					
	AX-94824733	3B	NA					

**Figure 10:**
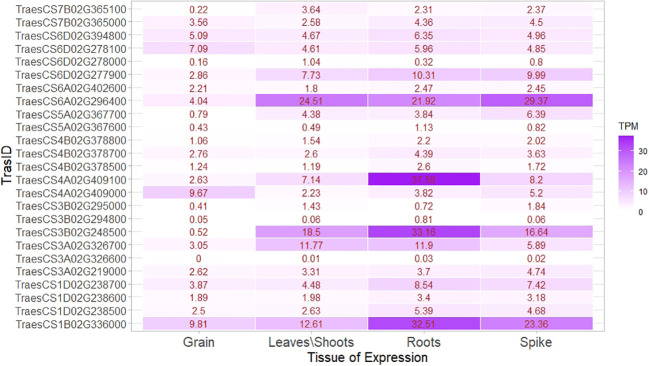
Expression heat map of identified genes in important tissues, that is, root, shoot/leaves, spikes, and grain.

An expression study of candidate genes using wheat expression data revealed several transcripts, that is, TraesCS4A02G409100, TraesCS6A02G296400, TraesCS3B02G248500, TraesCS1B02G336000 and TraesCS6D02G277900, which had very high expression in root and shoots, compared with grains and genes, and mainly belonged to MTAs linked to the TGW. The ranges of expression in root, shoot, spike, and grains were 0.03–37.58 TPM, 0.01–24.51 TPM, 0.02–29.37 TPM, and 0–9.18 TPM, respectively. Among the transcripts expressed in grains, TraesCS6D02G394800, TraesCS6D02G278100, TraesCS4A02G409000, and TraesCS1B02G336000 had higher expression than others. Transcript TraesCS1B02G336000 was linked with higher expression in both vegetative tissues and grain (**Figure 10**). The transcript producing secretory carrier membrane proteins (SCAMPs) was found to be important in subcellular localization and trafficking (Law et al., 2012).

## Discussion

Wheat has nutritional importance in combatting hidden hunger; however, the grain is deficient in iron and zinc. Environmental influence often affects the quality, quantity, and nutritional status of the grain because of environmental influences, such as heat and drought stress, during the growth phase (Sehgal et al., 2018). It is necessary to have a deep knowledge of the genomic region influencing grain iron and zinc content under drought and heat stress in order to develop specific varieties with a high micronutrient status along with a higher yield. Previously identified drought- and its component trait-related QTLs were utilized to improve drought tolerance of varieties through marker-assisted back crossbreeding (Sunilkumar et al., 2022). For such implications, the identification of a marker linked with the trait of interest is a prerequisite. Hence, the use of a diverse mapping panel for the identification of MTAs linked to GFeC, GZnC, and TKW in wheat grown under heat and drought stress along with control plants was attempted in this study.

Analysis of variance for all the studied traits (except GFeC under IR in 2021) in both years showed significant variation among the traits. The trait variations are a prerequisite for genetic studies and a breeding program of the nutrient status of the grain (Cu et al., 2020). Trait values of GFeC and GZnC were enhanced under drought and heat stress, as compared to the control, due to a lower yield, which is called the concentration effect (Oury et al., 2006; Morgounov et al., 2007; Fan et al., 2008; Liu et al., 2014). A greater yield lowers the grain nutrient content due to the dilution effect (Gupta et al., 2021). However, grain iron and zinc contents were higher in stress conditions, except for GZnC under RI in 2021 (39.24 mg/kg), compared with the control. Given that GFeC and GZnC are complex traits, with a quantitative pattern of inheritance and environmental influence, their expression levels might differ with the season, treatment, and soil nutrient condition, etc.

The coefficient of variation (CV) and broad-sense heritability for GZnC and TKW were higher compared with GFeC. TGW was having high heritability throughout the six environments followed by GZnC, which was similar to previous reports by Krishnappa et al., 2022. Heritability of GFeC was low to medium, having a wide range from 19.42% to 61.43%. Higher heritability indicates the greater contribution of genetic variance to the total variance and indicates the possibility of genetic improvement of traits through marker-assisted selection (Rathan et al., 2022). Inter-season correlation among TGW measured under three different treatments across two years had a strong positive correlation, indicating the higher heritability of the trait. Inter-season correlation for GFeC and GZnC was positive, but the strength was low, similar to a previous report on GZnC (Alomari et al., 2018); this might be because of environmental influence.

We observed that the Pearson correlation coefficient was positive between GFeC and GZnC under all the treatments (except RI in 2020), which was similar to the results reported by Liu et al. (2014); Rathan et al. (2022); Krishnappa et al. (2022). However, Cu et al. (2020) reported both positive and non-significant correlations between GFeC and GZnC in matured and immature grains. TGW had no correlation with GFeC and GZnC in 2020–2021, whereas it showed a positive correlation with GFeC in 2021–2022. Correlation between TGW and GFeC showed positive (Rathan et al., 2022), negative, and no correlations (Cu et al., 2020) in previous studies. Traits harbored by additive gene action and with positive correlation can be improved together efficiently despite environmental influences (Borah et al., 2018).

The use of structured populations influences the identification of MTAs through GWASs (Pritchard et al., 2000). To nullify this effect, population structure is used as a covariate in the analysis. The present material had three subpopulations, as identified by marker-based PCA. PCA is one of the popular approaches for inferring the population structure of the genome-wide association panel using high-density SNPs (Abraham and Inouye, 2014; Devate et al., 2022a). A diversity tree, based on the genetic distance, confirms that there was sufficient diversity by having three main clusters and many subclusters branching further. The grouping pattern seemed to correlate with the origin of materials. All the core-set lines in the GWAS panel were grouped in one group and the varieties of Indian origin were in another.

The linkage disequilibrium (LD) decay over genetic distance in a population determines the density of marker coverage needed to perform GWASs. A faster LD decay indicates the requirement of a higher marker density to capture the markers close enough to the causal loci (Flint-Garcia et al., 2003). In the present study, a large LD block size was found with 3.49 Mb for the whole genome. The LD for the subgenomes was found to be 2.48 Mb, 4.29 Mb, and 3.82 Mb for A, B, and D genomes, respectively. Similarly, a large LD block size of 4.4 Mb was observed by Pang et al., 2020. In the current study, a low rate of LD decay was observed for the B genome followed by D and A, whereas faster LD decay (Ledesma-Ramírez et al., 2019), as well as slow decay in the D genome, were reported in previous studies (Ogbonnaya et al., 2017; Jamil et al., 2019; Li et al., 2019; Pang et al., 2020; Devate et al., 2022a). The LD may vary in different populations because of population size, genetic drift, admixtures, selection, mutation, non-random mating, pollination behavior, and recombination frequency (Gupta et al., 2005; Vos et al., 2017).

A genome-wide association study was carried out with the BLINK model under GAPIT, which is presumed to be superior for identifying QTNs and avoiding false positives to decipher true associations (Huang et al., 2019). A total of 36 MTAs were identified to be linked to the studied traits at a *p*-value of<0.0001. However, to enhance the stringency of selection to avoid false positives, a Bonferroni correction was applied. A total of 16 stringent markers were found: five for GFeC, one for GZnC, and 10 for TGW. Bonferroni correction is a method to counteract the multiple comparisons problem to reduce type 1 error, i.e., false positives (Kaler and Purcell, 2019).

Recent advances in wheat genome sequencing and annotation made it possible to decipher the candidate genes in the genomic region identified with GWAS, which may be responsible for the variation of the grain nutrient content directly or indirectly by influencing the nutrient metabolism (Cu et al., 2020). Candidate genes located near 100-kb regions of identified MTAs were tabulated with their transcript ID and the probable proteins they code for. Tissue specific expression of genes, responsible for stress management and nutrient content in the grains were identified in the genomic region of linked SNP markers. Stress-related expression of genes in roots, as well as in seed, were identified based on the expression study using the Wheat Expression Database and the Expression Atlas.

Marker trait associations (MTAs) with GFeC were found on chromosomes 3A, 3B, 4B, 6B, and 7D; there have been similar reports on chromosome 3B (Crespo-Herrera et al., 2017; Liu et al., 2019; Cu et al., 2020; Liu et al., 2021; Krishnappa et al., 2022), whereas the rest of the MTAs were novel to this study. The grain iron content-linked SNP marker AX-94877284 was identified to be present near the candidate regions coding for NB-ARC, the winged helix-like DNA-binding domain, and the synaptotagmin-like mitochondrial-lipid-binding domain. The coding regions were identified through *in silico* analysis. Furthermore, the expression study revealed their expression in the grain as well as in the root, and they are an integral part of the membrane, acting as a lipid transporter. These three important proteins have a crucial role in plant disease resistance (Van Ooijen et al., 2008), iron deficiency response (Colangelo and Guerinot, 2004), and homeostasis during abiotic stress (Ruiz-Lopez et al., 2021). As wheat is a hexaploid with three homeologous genomes there is a high chance that similar genes on the respective homeologous chromosomes would be found. An elaborate study of iron deficiency-specific clone 3 (Ids3)-like genes in hexaploid wheat was conducted by Mathpal et al., 2018. The authors found an ortholog on the telomeric region of chromosome 7A and its homolog ranged from 78% to 88% on chromosomes 7D and 7B. Similarly, we found a novel region related to GFeC on chromosome 7B linked to MTA AX-94393306. The region was found to be present near the gene region that encodes the HAUS augmin–like complex subunit. This subunit has an influence on microtubule development during mitosis (Uehara et al., 2009) and on the P-loop-containing nucleoside triphosphate hydrolase whose homologs have the property to bind divalent cation (Tan et al., 2010). Iron has both divalent and trivalent valency. However, the majority of its uptake and assimilation only take place in its divalent status (Tsai and Schmidt, 2017). There is evidence to influence transporters on more than one kind of ions (Pinilla-Tenas et al., 2011; Wang et al., 2012). The candidate region coding for the tyrosinase copper-binding domain, which performs a metal-binding activity (Solano, 2018), was identified in the vicinity of the marker AX-94565216. SNP marker AX-94850629 was linked with the candidate genes coding for the serine–threonine/tyrosine-protein kinase catalytic domain and the bifunctional inhibitor/plant lipid transfer protein/seed storage helical domain. The first gene influences plant responses to stress signals and developmental processes involving modifications in protein Tyr phosphorylation (Shankar et al., 2015). The second gene is a seed storage proteins homolog such as napin from *Brassica napus* (Rico et al., 1996) and 2S albumin from *Ricinus communis* (Pantoja-Uceda et al., 2003). Furthermore, their cellular function was found to be protein phosphorylation and regulation of protein kinase activity. A differential regulation of the transcript during drought and heat stress (Gahlaut et al., 2020) was maintained by a candidate gene near AX-95168777, i.e., C-5 cytosine methyltransferase under the RI condition. Differential regulation of genes may have an influence on nutrient homeostasis in plants.

Stringent selection for MTAs left only one marker AX-95095792 on chromosome 4B linked with grain zinc content. The candidate region is mainly responsible for gene regulation and translation-related activities. Cyclophilin-type peptidyl-prolyl cis–trans isomerase domain, tRNA/rRNA methyltransferase, and SpoU-type proteins were reported in the candidate region. They have functions in the isomerization of the prolyl–peptide bond (Singh et al., 2021) and tRNA modification linked to protein synthesis (Hori, 2017). However, for GZnC, as a quantitative trait, the study revealed its low level of expression in the root as well as the grain and demonstrated it to be a minor gene, which is a common feature of quantitative traits governed by many genes. As housekeeping genes are expressed regularly and have importance in various metabolic and physiological activities of plants, this region might have an indirect influence on the zinc content of grains.

A total of eight unique MTAs were identified for TGW located on chromosomes 1B, 1D, 2B, 3B, 4A, 5A, 6A, and 6D. Similarly, previous studies reported TGW-related QTLs/MTAs on chromosomes 6A (Godoy et al., 2018; Ward et al., 2019; Goel et al., 2019), 5A (Liu et al., 2019; Krishnappa et al., 2022), and 1D (Goel et al., 2019). Stably identified MTA for TGW under different treatments and combined BLUP, AX-94926681 located on chromosome 6A, was identified with transcript TraesCS6A02G402600. The locus codes for three major enzymes ribonuclease H-like and exonuclease and RNase T/DNA polymerase III were identified and are related to the enzymes responsible for nucleic acid metabolism, replication, homologous recombination, DNA repair, transposition (Majorek et al., 2014), epigenetic changes at RNAPIII (Hummel and Liu, 2022), and the repair of double-stranded breaks in *Arabidopsis thaliana* (Peralta-Castro et al., 2020). Their presence as an integral part of the membrane and property of DNA binding further confirms the results based on gene ontology. Another marker, AX-94888039, located in the region responsible for stress management, had candidate genes expressed in the cytoplasm and nucleus. These genes encode chaperonin Cpn60/GroEL, which is involved in abiotic stress-induced expression (Nagaraju et al., 2021), and the zinc finger FYVE/PHD type, which has a role in abiotic stress tolerance by regulating sodium and potassium homeostasis, reactive oxygen species scavenging, and osmotic potential (Zang et al., 2016). Chaperonin Cpn60/GroEL was found to have a very high expression of up to 37.58 TPM in root tissue under abiotic stress conditions. Two MTAs were found to be linked to TGW under RI conditions, that is, AX-94546067 and AX-94981340. Their transcripts code for the NUDIX hydrolase domain and the ABC transporter-like, ATP-binding domain, with their function being related to plant immune responses as found in *Arabidopsis thaliana* (Fonseca and Dong, 2014), the negative regulator in response to drought stress in peach (He et al., 2022), and the transport of substrates into and out of the cytoplasm in plants (Locher, 2016). A secretory pathway, subcellular localization, and trafficking-related secretory carrier membrane proteins (SCAMPs) were found in the region of SNP AX-94488007. The location of marker AX-94488007 was in the seventh intronic region of the gene coding for secretory carrier membrane proteins (SCAMPs) and was found to have very high levels of expression in the root (i.e., 32.51 TPM), as well as in grain (i.e., 9.81 TPM), suggesting the importance of this gene in grain filling. It was also found to have higher levels of expression under abiotic stress conditions of up to 17.81 TPM from the wheat expression database. Photosynthate mobilization and accumulation are key factors that determine the source–sink relationship and, hence, may influence grain filling (Chang and Zhu, 2017). However, further study is required to elucidate the role of candidate genes in determining the TGW. The transcript TraesCS5A02G367700 was linked with a TGW-related SNP, AX-94937080. This was found to code for a protein kinase domain and a G-protein beta WD-40 repeat. WD40 repeat proteins were found to have a role in plant cell wall formation (Guerriero et al., 2015), a unique property of the plant cell. The genomic region of AX-95189725 was located in the third exonic region of gene coding for ankyrin and pentatricopeptide repeat proteins, which are found to be responsible for drought and salt tolerance in *Arabidopsis* and soybean (i.e., gene GmANK114) (Zhao et al., 2020), and pollen development in rice (Zhang et al., 2020). Most of the genes identified in the candidate region were found to play a crucial role in plant development. Many of their homologs have been studied extensively in other species, giving us a broad idea of their role. Expression analysis and gene ontology identified genes with higher expression along with their biological role in the cell organelles. Further study of each of the identified locations could be carried out to gain a better understanding of the genomic region and its potential role in a breeding program.

## Conclusion

The development of biofortified wheat varieties through the transfer of genomic regions associated with higher concentrations of micronutrients in grain will aid in the health of millions of malnourished people suffering from hidden hunger. Genetic biofortification is easy, economical, and sustainable in the long term. Identification of genomic regions using linked markers is a prerequisite to transfer them into the popular cultivars to develop a variety with a higher grain nutrient content. The association mapping panel used in this study with 193 wheat genotypes revealed that GFeC, GZnC, and TGW are complex traits that are quantitatively inherited and strongly influenced by abiotic stress, such as drought and heat stress. The significant positive correlation between the GFeC and GZnC, as well as their high heritability, suggests that simultaneous improvement of both traits can be possible. Five of the 16 stringent MTAs identified were linked to GFeC, one to GZnC, and 10 to TGW, and were located near novel candidate genes that have a direct or indirect effect on traits. Several putative candidate genes identified were found to encode proteins that have important molecular functions in plant ionic balance, protein and enzyme metabolism, and abiotic stress responsiveness, etc. Furthermore, identified MTAs could be subjected to validation and utilized in marker-assisted breeding programs to create biofortified varieties.

## Data availability statement

All the raw data used in this study is available at https://doi.org/10.5061/dryad.0cfxpnw6c.

## Author contributions

PS, NJ, GS, and HK conceptualized the investigation and edited the manuscript. ND conducted the investigation and prepared the draft of the manuscript. ND, CM, and KM generated the phenotypic data. ND, HK, PS, SS, and DC contributed to the generation of genotyping data. ND and HK did the statistical and GWAS analysis. All authors contributed to the article and approved the submitted version.
