# Supplementary material for: Genetic dissection of marker trait associations for grain micro-nutrients and thousand grain weight under heat and drought stress conditions in wheat
Source: Front Plant Sci. 2023 Jan 16;13:1082513. doi: 10.3389/fpls.2022.1082513 (PMC9885108; doi:10.3389/fpls.2022.1082513)
Supplement: Supplementary file 1 [file DataSheet_1.zip › Data Sheet 1 (24)/Supplementary Figure 1.docx]

Supplementary fig 1: Inter season and treatment correlation for each traits

1. Correlation among GFeC data across the season and treatment


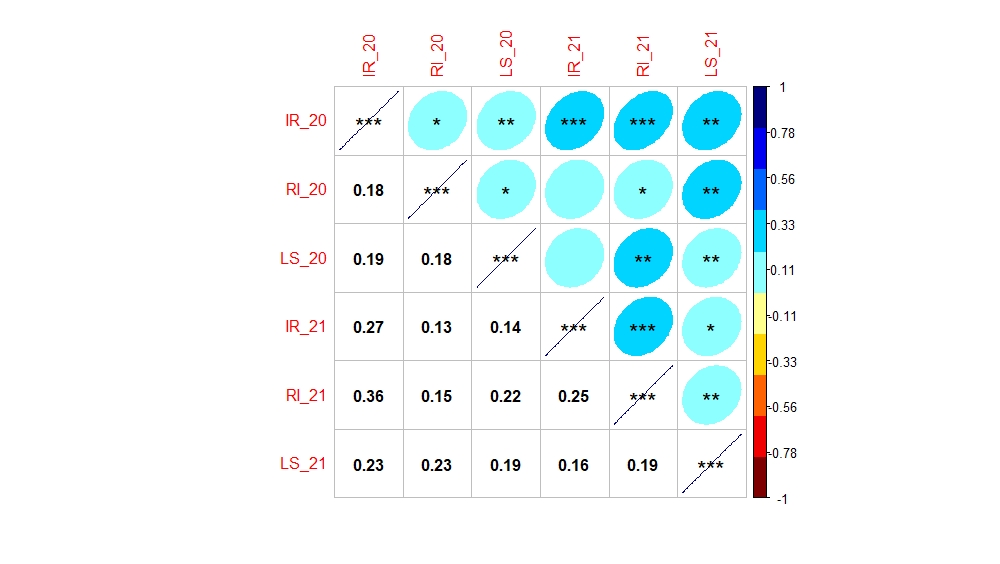


1. Correlation among GZnC data across the season and treatment

**
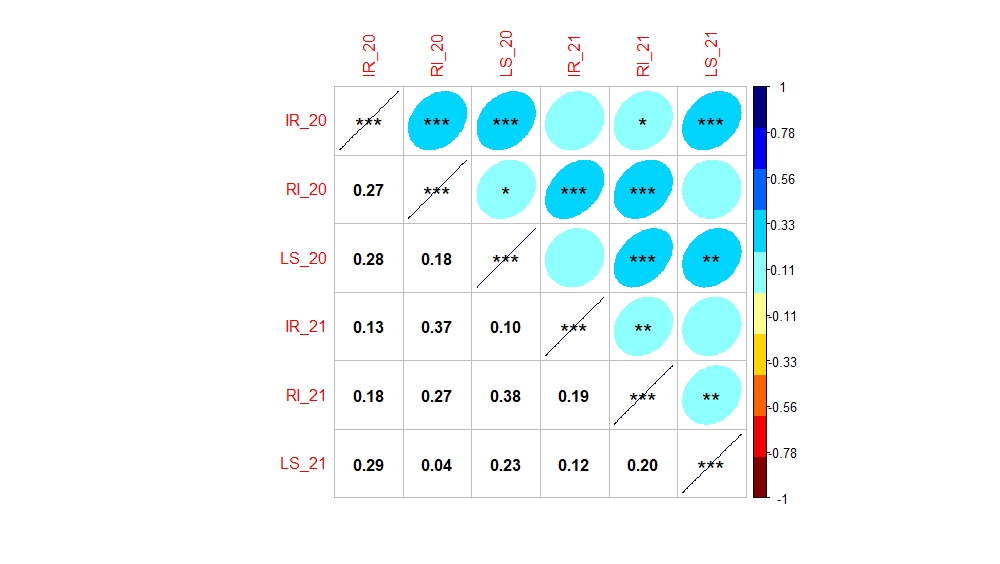
**

1. Correlation among TGW data across the season and treatment

**
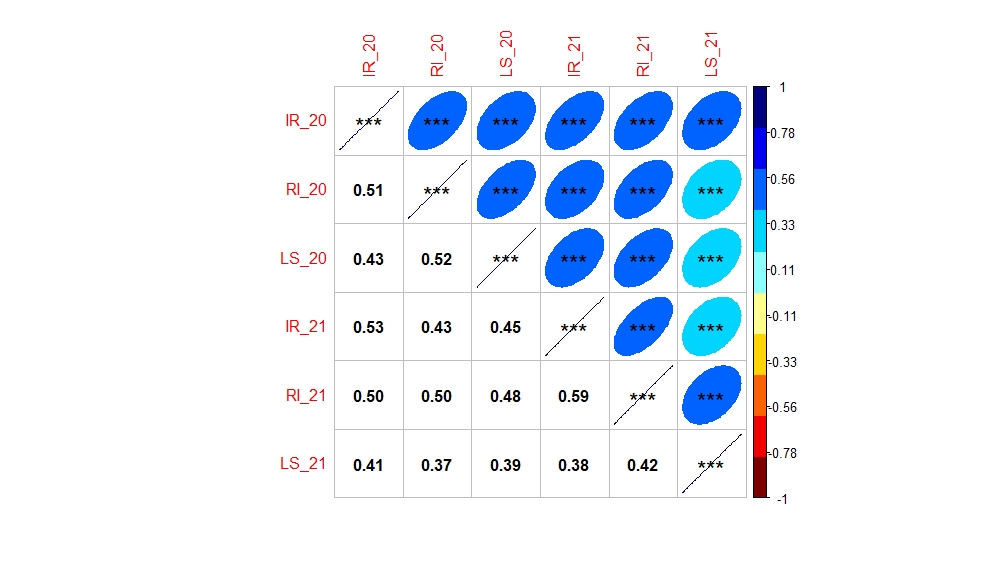
**
