# Supplementary material for: Genetic dissection of marker trait associations for grain micro-nutrients and thousand grain weight under heat and drought stress conditions in wheat
Source: Front Plant Sci. 2023 Jan 16;13:1082513. doi: 10.3389/fpls.2022.1082513 (PMC9885108; doi:10.3389/fpls.2022.1082513)
Supplement: Supplementary file 1 [file DataSheet_1.zip › Data Sheet 1 (24)/Supplementary table 3.docx]

Supplementary table 2: List MTAs identified at threshold p value 0.0001 (over all 51, 36 after removing the duplicates)

| Sr. No | Trait | SNP | Chromosome | Position | P.value | LOD |
| --- | --- | --- | --- | --- | --- | --- |
| 1 | FE_IR_20 | AX-94877284 | 6D | 467912559 | 5.77E-09 | 8.238789 |
| 2 | FE_IR_20 | AX-94625273 | 5D | 381035922 | 8.33E-05 | 4.079245 |
| 3 | FE_IR_BLUP | AX-95253818 | 3D | 363603208 | 1.11E-05 | 4.95344 |
| 4 | FE_IR_BLUP | AX-94850629 | 3B | 473694803 | 3.84E-05 | 4.415159 |
| 5 | FE_RI_20 | AX-94865230 | 5D | 288358211 | 7.33E-05 | 4.134775 |
| 6 | FE_RI_BLUP | AX-95168777 | 3A | 401710412 | 1.29E-07 | 6.890502 |
| 7 | FE_LS_20 | AX-94393306 | 7B | 628451664 | 3.39E-06 | 5.470092 |
| 8 | FE_LS_20 | AX-94485883 | 6B | 664407511 | 5.72E-05 | 4.242969 |
| 9 | FE_LS_21 | AX-94565216 | 3A | 571919080 | 8.49E-08 | 7.071115 |
| 10 | FE_LS_BLUP | AX-94393306 | 7B | 628451664 | 1.74E-11 | 10.75943 |
| 11 | FE_CBLUP | AX-94850629 | 3B | 473694803 | 4.63E-09 | 8.334826 |
| 12 | ZN_IR_20 | AX-95095792 | 4B | 660465268 | 1.17E-07 | 6.929969 |
| 13 | ZN_IR_21 | AX-95231040 | 3A | 106397883 | 5.34E-05 | 4.272689 |
| 14 | ZN_RI_20 | AX-94876345 | 3A | 738134025 | 4.85E-05 | 4.313849 |
| 15 | ZN_LS_21 | AX-94384140 | 5A | 659165855 | 5.04E-05 | 4.297886 |
| 16 | ZN_LS_21 | AX-95197417 | 5B | 13701543 | 8.49E-05 | 4.071129 |
| 17 | TGW_IR_20 | AX-94926681 | 6A | 610406742 | 3.96E-08 | 7.401876 |
| 18 | TGW_IR_20 | AX-94888039 | 4A | 681717562 | 4.15E-07 | 6.381996 |
| 19 | TGW_IR_20 | AX-95133680 | 1D | 328102224 | 7.11E-07 | 6.148171 |
| 20 | TGW_IR_20 | AX-94937080 | 5A | 567917351 | 5.23E-06 | 5.281828 |
| 21 | TGW_IR_21 | AX-94546067 | 6A | 530046844 | 1.15E-05 | 4.937796 |
| 22 | TGW_IR_21 | AX-95189725 | 6D | 386433138 | 2.24E-05 | 4.649788 |
| 23 | TGW_IR_21 | AX-94513729 | 7D | 74409987 | 3.76E-05 | 4.424465 |
| 24 | TGW_IR_21 | AX-94540984 | 2B | 493986775 | 5.05E-05 | 4.296387 |
| 25 | TGW_IR_21 | AX-95123732 | 6B | 605552198 | 7.63E-05 | 4.117527 |
| 26 | TGW_IR_21 | AX-94575638 | 6A | 531101078 | 8.13E-05 | 4.089698 |
| 27 | TGW_IR_21 | AX-95099328 | 2B | 490344182 | 8.17E-05 | 4.087733 |
| 28 | TGW_IR_BLUP | AX-94926681 | 6A | 610406742 | 5.05E-10 | 9.296763 |
| 29 | TGW_IR_BLUP | AX-94488007 | 1B | 562893270 | 3.29E-07 | 6.483375 |
| 30 | TGW_IR_BLUP | AX-94937080 | 5A | 567917351 | 5.12E-07 | 6.290645 |
| 31 | TGW_IR_BLUP | AX-95189725 | 6D | 386433138 | 1.98E-06 | 5.702482 |
| 32 | TGW_IR_BLUP | AX-94513729 | 7D | 74409987 | 3.43E-05 | 4.464104 |
| 33 | TGW_IR_BLUP | AX-94888039 | 4A | 681717562 | 4.70E-05 | 4.327726 |
| 34 | TGW_RI_20 | AX-94926681 | 6A | 610406742 | 4.42E-05 | 4.35481 |
| 35 | TGW_RI_20 | AX-95012703 | 2D | 634124454 | 9.17E-05 | 4.037677 |
| 36 | TGW_RI_21 | AX-94926681 | 6A | 610406742 | 5.35E-12 | 11.27166 |
| 37 | TGW_RI_21 | AX-94546067 | 6A | 530046844 | 1.79E-07 | 6.746621 |
| 38 | TGW_RI_21 | AX-94981340 | 3B | 395914598 | 6.07E-07 | 6.217013 |
| 39 | TGW_RI_21 | AX-94950047 | 2B | 232916533 | 1.90E-06 | 5.720823 |
| 40 | TGW_RI_21 | AX-94686023 | 7A | 583838327 | 1.34E-05 | 4.872312 |
| 41 | TGW_RI_BLUP | AX-94926681 | 6A | 610406742 | 8.73E-11 | 10.05904 |
| 42 | TGW_LS_21 | AX-95117670 | 7D | 519260823 | 6.08E-05 | 4.216342 |
| 43 | TGW_LS_BLUP | AX-94926681 | 6A | 610406742 | 7.50E-08 | 7.125002 |
| 44 | TGW_LS_BLUP | AX-94702774 | 7A | 126907819 | 8.20E-05 | 4.086003 |
| 45 | TGW_LS_BLUP | AX-94574610 | 7A | 126907922 | 8.52E-05 | 4.069345 |
| 46 | TGW_CBLUP | AX-94926681 | 6A | 610406742 | 8.56E-11 | 10.06758 |
| 47 | TGW_CBLUP | AX-94824733 | 3B | 195361587 | 5.82E-08 | 7.235218 |
| 48 | TGW_CBLUP | AX-94484728 | 2B | 214004581 | 1.25E-05 | 4.904172 |
| 49 | TGW_CBLUP | AX-94702774 | 7A | 126907819 | 6.03E-05 | 4.219864 |
| 50 | TGW_CBLUP | AX-94816345 | 6A | 531101113 | 7.33E-05 | 4.13518 |
| 51 | TGW_CBLUP | AX-94888039 | 4A | 681717562 | 9.51E-05 | 4.021986 |
